# Variations in dosimetric distribution and plan complexity with collimator angles in hypofractionated volumetric arc radiotherapy for treating prostate cancer

**DOI:** 10.1002/acm2.12249

**Published:** 2018-01-11

**Authors:** Ming‐Hsien Li, Sheng‐Fang Huang, Chih‐Chieh Chang, Jang‐Chun Lin, Jo‐Ting Tsai

**Affiliations:** ^1^ Department of Radiation Oncology Shuang Ho Hospital Taipei Medical University Taipei Taiwan China; ^2^ School of Medicine College of Medicine Department of Radiology Taipei Medical University Taipei Taiwan China

**Keywords:** arc radiotherapy, collimator angle, modulation complexity score

## Abstract

**Purpose:**

Hypofractionated radiotherapy can reduce treatment durations and produce effects identical to those of conventionally fractionated radiotherapy for treating prostate cancer. Volumetric arc radiotherapy (VMAT) can decrease the treatment machine monitor units (MUs). Previous studies have shown that VMAT with multileaf collimator (MLC) rotation exhibits better target dose distribution. Thus, VMAT with MLC rotation warrants further investigation.

**Methods and materials:**

Ten patients with prostate cancer were included in this study. The prostate gland and seminal vesicle received 68.75 and 55 Gy, respectively, in 25 fractions. A dual‐arc VMAT plan with a collimator angle of 0° was generated and the same constraints were used to reoptimize VMAT plans with different collimator angles. The conformity index (CI), homogeneity index (HI), gradient index (GI), normalized dose contrast (NDC), MU, and modulation complexity score (MCS_V_) of the target were analyzed. The dose–volume histogram of the adjacent organs was analyzed. A Wilcoxon signed‐rank test was used to compare different collimator angles.

**Results:**

Optimum values of CI, HI, and MCS_V_ were obtained with a collimator angle of 45°. The optimum values of GI, and NDC were observed with a collimator angle of 0°. In the rectum, the highest values of maximum dose and volume receiving 60 Gy (V_60 Gy_) were obtained with a collimator angle of 0°, and the lowest value of mean dose (D_mean_) was obtained with a collimator angle of 45°. In the bladder, high values of D_mean_ were obtained with collimator angles of 75° and 90°. In the rectum and bladder, the values of V_60 Gy_ obtained with the other tested angles were not significantly higher than those obtained with an angle of 0°.

**Conclusion:**

This study found that MLC rotation affects VMAT plan complexity and dosimetric distribution. A collimator angle of 45° exhibited the optimal values of CI, HI, and MCSv among all the tested collimator angles. Late side effects of the rectum and bladder are associated with high‐dose volumes by previous studies. MLC rotation did not have statistically significantly higher values of V_60 Gy_ in the rectum and bladder than did the 0° angle. We thought a collimator angle of 45° was an optimal angle for the prostate VMAT treatment plan. The findings can serve as a guide for collimator angle selection in prostate hypofractionated VMAT planning.

## 1 INTRODUCTION

Volumetric modulated arc therapy (VMAT) has become a standard delivery option for prostate radiotherapy because it has a shorter delivery time and requires fewer monitor units (MUs) than does step‐and‐shoot intensity modulated radiotherapy (IMRT). In addition, some studies have revealed that prostate VMAT and IMRT exhibit comparable target coverage and normal tissue (bladder, rectum, and femoral heads) sparing.^1^, ^2^, ^3^ In radiotherapy, the sensitivity of tumors to changes in fractionation can be quantified in terms of the α/β ratio. The α/β values for most human tumors are high (typically 10 Gy). Recent studies have suggested that adenocarcinomas of the prostate gland, with a low average α/β ratio of <2 Gy, differ from most other malignancies.^4^, ^5^ The treatment of tumors with low α/β ratios through hypofractionated IMRT with a high dose per fraction requires a short duration and exhibits efficacy and toxicity levels similar to those of conventionally fractionated IMRT.^6^, ^7^, ^8^ However, increasing the dose per fraction requires a higher number of MUs and a longer treatment duration per fraction. For the same treatment plan, hypofractionated VMAT requires fewer MUs and a shorter treatment time per fraction than does hypofractionated IMRT and therefore is a viable, safe, and comfortable treatment technique for prostate cancer.^9^

VMAT technology simultaneously combines gantry rotation speed, multileaf collimator (MLC) motion, and dose rate modulation. In general, the complex target shapes, volumes of targets, and multiple prescribed dose levels require the use of two or more VMAT arcs to improve dosimetric distribution.^10^, ^11^ MLC is the most suitable tool for beam shaping and is designed to have a tongue‐and‐groove shape on the side of each leaf for reducing interleaf radiation leakage. However, transmission through the leaves remains nonuniform; thus, MLC rotation in the VMAT can minimize interleaf radiation leakage. In addition, several studies have reported that MLC rotation improves spatial resolution and target dose distribution.^12^, ^13^, ^14^, ^15^, ^16^, ^17^

In the present study, the dosimetric distribution and plan complexity obtained using various collimator angles (0°, 15°, 30°, 45°, 60°, 75°, and 90°) for dual‐arc hypofractionated regimens of VMAT with simultaneous integrated boost VMAT (SIB‐VMAT) in patients with prostate cancer. This study identified the optimum collimator angles for optimizing dosimetric distribution for planning target volume (PTV), sparing of organs at risk (OARs), and plan complexity. The findings of this study could help planners to select appropriate collimator angles to obtain optimum results.

## 2 METHODS AND MATERIALS

### 2.A Patient selection and planning criteria

This study was designed to compare plan complexity and dose distribution among several collimator angles (0°, 15°, 30°, 45°, 60°, 75°, and 90°). Ten patients with prostate cancer without pelvic lymph node enlargement were recruited for this study. The clinical target volume (CTV) of the prostate gland (CTV_P_) consisted of the entire prostate gland and that of the seminal vesicle (CTV_S_) consisted of the entire seminal vesicle. The planning target volume (PTV) of the prostate gland (PTV_P_) consisted of the CTV_P_ and a 5‐mm margin (except at the CTV–rectum interface, where a 3‐mm margin was used). Identical criteria were applied to create the planning target volume of the seminal vesicle (PTV_S_).

In order to reduce the complexity of radiation treatment planning, we used single‐phase hypofractionated SIB‐IMRT regimen published by Maurizio, with the exception that prophylactic irradiation of the pelvic lymph area was not performed in this study.^18^ According to the plan, the PTV_P_ and PTV_S_ received 68.75 Gy (2.75 Gy per fraction) and 55 Gy (2.2 Gy per fraction), respectively, in 25 fractions. Acceptable plans were defined as the prescribed dose covering at least 95% of the PTV. The OARs dose–volume constraints were as follows: rectum, V_52 Gy_ <35% and V_61 Gy_ <25%; bladder, V_45 Gy_ <50%; and femoral heads V_50 Gy_ <10%.

### 2.B VMAT plan and treatment delivery

SIB‐VMAT plans were generated using the Pinnacle treatment planning system (Philips, Version 9.8.0, Fitchburg, WI, USA) for 10‐MV beams from an Elekta Precise Linear Accelerator (LINAC; Elekta Ltd., Crawley, UK) and optimized using the direct machine parameter optimization algorithm. Integrated MLC consists of 40 opposed pairs of leaves, with a projected width of 1 cm at the isocenter. The total leaf travel distance is 32.5 cm and jaws cover a full 40 × 40 cm^2^ field. No leaf interdigitation is allowed and the minimum gap between the opposed leaves and opposed adjacent leaves is 0.5 cm. All calculations were performed using an adaptive convolve with a calculation grid spacing of 0.3 cm. Each plan resulted from dual‐arc with a gantry and rotation of 181°–180°–181°. We optimized the dual‐arc VMAT treatment plan with the collimator angle set to 0° and then used the same constraints to reoptimize VMAT plans using different values for collimator angles (15°, 30°, 45°, 60°, 75°, and 90°) for each patient. A maximum delivery time of 300 s/arc and a final gantry spacing of 4° were used during the optimization. The constraint leaf motion was set to 0.33 cm/deg. The maximum leaf velocity was 2 cm/s, the maximum gantry velocity was 6 deg/s, and the maximum variable dose rate was 600 MU/min.

### 2.C Dosimetric evaluation

For dosimetric comparison, 10‐patient average values of parameters such as the conformity index (CI), homogeneity index (HI), gradient index (GI), and normalized dose contrast (NDC) of the PTV for collimator angles were used.

Based on the definition in the International Commission on Radiation Units and Measurements report 62, CI refers to the volume of the target receiving the prescribed dose divided by the volume of the PTV_P_, and has an optimal value of 1.

(1) $$CI=V_{100\%}/PTV_{P}$$

The HI is defined as the dose received by 2% of the PTV minus the dose received by 98% of the PTVp divided by the prescribed dose (its optimal value is 0). The HI is calculated as follows:

(2) $$HI=(D_{2\%}-D_{98\%})/Prescribed\;dose$$

The GI is defined as the ratio of the volume covered by 50% of the prescribed dose to the treated volume of the PTV_P_. The GI is calculated as follows:

(3) $$GI=V_{50\%}/V_{100\%}$$

The delivery of a high dose to a high‐dose target volume unavoidably increases the dose to the surrounding low‐dose target volume. To assess the quality of an SIB plan, the NDC is used to compare the dose gradient. Dose contrast (DC) is defined as the mean dose of PTV_P_ divided by the mean dose of PTV_S_.^19^ The ideal DC of an SIB plan is the ratio of the prescribed dose of PTV_P_ to the prescribed dose of PTV_S_. Therefore, the ratio of the actual DC to the ideal DC is defined as NDC (its optimal value is 1).

### 2.D Modulation complexity score for VMAT

For each of the 70 SIB‐VMAT plans, the modulation complexity score of the SIB‐VMAT plans (MCS_V_) was calculated from the DICOM RT files. The modulation complexity score was originally proposed by McNiven for fixed‐beam IMRT as a normalized sum over all the segments of the aperture area variability and leaf sequence variability.^20^ Masi modified the score to suit VMAT plans by substituting the control points of the arc with segments.^21^ As in the original definition, the MCS_V_ has values ranging from 0 to 1. MCS_V_ = 1 indicates no modulation, and can be represented by an arc with a fixed rectangular aperture without any movement along the arc. When modulation increases, the MCS_V_ decreases. The total number of MUs for each plan was a crucial indicator of plan complexity and was included in the analysis.

### 2.E Statistical analysis

The Wilcoxon signed‐rank test was used for multiple comparison of the target parameters and critical organs at different collimator angles. *P *≤* *0.05 was defined as statistically significant.

## 3 RESULTS

**Figure 1:**
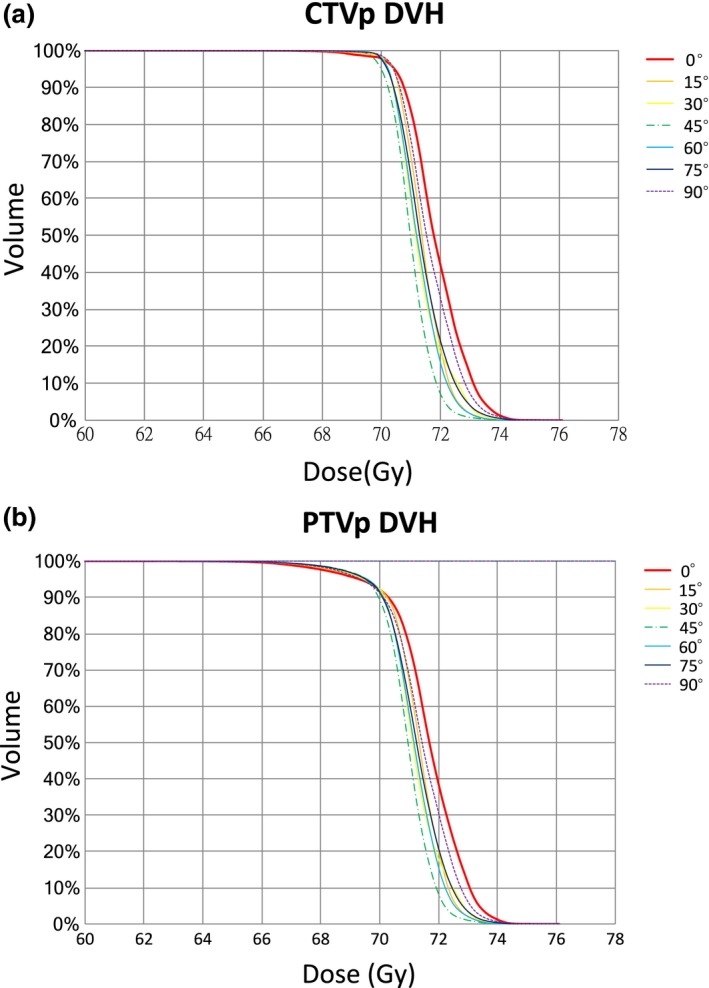
(a) Average dose–volume histogram of the CTV_P_. (b) Average dose–volume histogram of the PTV_P_.

**Figure 2:**
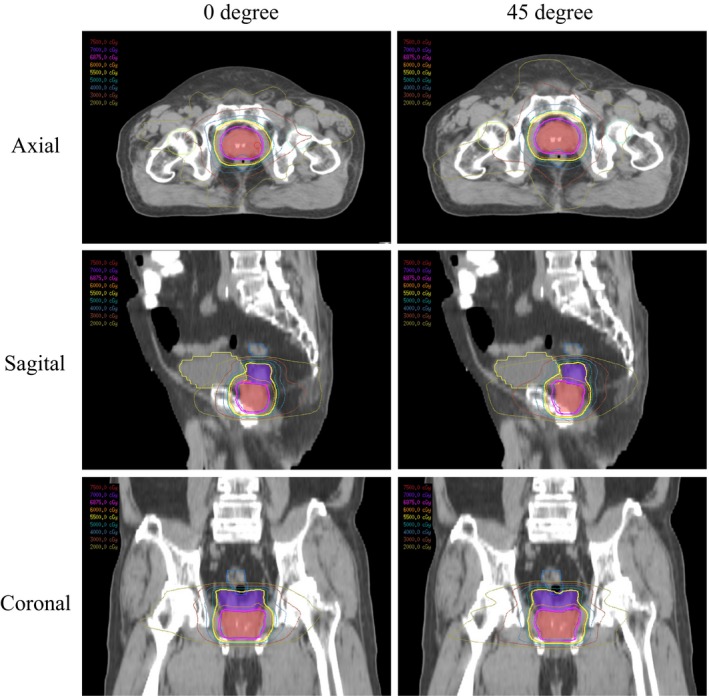
The dose distribution of prostate plans at the collimator angles of 0° and 45°.

The patient characteristics are presented in Table 1. The target and OARs volumes are summarized in Table 2. The dosimetric results of PTV for all studied collimator angles are summarized in Table 3 and the *P* values are summarized in Table 4. Comparisons of the PTV dosimetric results are summarized in Table 5. Average accumulated dose–volume histogram (DVH) of the CTV_P_ and the PTV_P_ is shown in Fig. 1. Figure 2 shows the dose distribution of prostate plans at the collimator angles of 0° and 45°. Table 3 reveals that a collimator angle of 0° had the highest CI value among all the tested angles. The values of CI and HI at a collimator angle of 45° was significantly close to the optimal value than did the 0° angle. Additionally, the 45° angle exhibited the lowest value of V_107%_ (0.35 cm^3^). The collimator angle of 0° exhibited the most inferior value of HI and the highest value of V_107%_ (3.29 cm^3^). However, the lowest value of V_50%_ and the optimal value of GI were obtained with the 0° angle. The highest value of V_50%_ was obtained with a collimator angle of 75° and the most inferior value of GI was observed with a collimator angle of 90°. The NDC values at collimator angles of 0° and 15° were nearly 1, and at all other tested angles, the NDC values were relatively inferior to the values at 0°.

**Table 1:** Patient characteristics

Patient No.	Age	TNM	Gleason score	Initial PSA (ng/mL)
1	78	T3aN0M0	7	51.21
2	76	T2cN0M0	8	37.03
3	65	T2cN0M0	7	8.82
4	81	T2cN0M0	7	38.48
5	75	T2cN0M0	7	12.35
6	71	T2cN0M0	7	47.19
7	74	T2cN0M0	7	87.91
8	68	T2cN0M0	10	28.76
9	83	T2cN0M0	6	44.00
10	87	T3aN0M0	9	43.80

**Table 2:** The volume characteristics for the target and the OARs (*n* = 10, cm^3^)

	Mean ± SD	Minimum	Maximum
Prostate	44.54 ± 23.23	24.51	101.85
PTV_P_	86.11 ± 35.34	53.75	171.06
Rectum	38.77 ± 13.27	24.72	69.05
Bladder	88.48 ± 55.37	45.7	224.16
Right femoral head	64.61 ± 11.28	45.43	80.61
Left femoral head	64.48 ± 10.29	46.34	81.41

**Table 3:** Dosimetric results of PTV (Mean ± SD, *n* = 10)

Parameters	Collimator angles						
	0°	15°	30°	45°	60°	75°	90°
V100% (%)	95.65 ± 1.08	96.08 ± 137	96.54 ± 1.38	96.83 ± 1.50	96.71 ± 1.55	96.67 ± 1.51	95.92 ± 1.07
V107% (cm^3^)	3.29 ± 5.18	0.53 ± 0.48	0.59 ± 1.10	0.35 ± 0.37	0.66 ± 0.98	1.21 ± 1.29	2.56 ± 4.20
V95% (cm^3^)	231.55 ± 312.14	130.65 ± 43.02	129.20 ± 42.14	129.05 ± 41.69	131.01 ± 42.81	131.69 ± 44.06	129.65 ± 43.18
V50% (cm^3^)	571.01 ± 119.16	583.03 ± 14.76	588.24 ± 137.72	586.97 ± 140.20	603.13 ± 150.11	604.63 ± 140.77	598.78 ± 136.77
D2% (cGy)	7336.07 ± 77.13	7297.26 ± 51.46	7280.07 ± 60.62	7273.51 ± 33.67	7290.31 ± 59.71	7313.94 ± 57.81	7337.70 ± 64.00
D98% (cGy)	6790.44 ± 69.75	6823.27 ± 72.61	6846.30 ± 70.61	6860.29 ± 62.37	6851.01 ± 69.63	6854.72 ± 67.23	6824.95 ± 48.84
Dmean (cGy)	7170.71 ± 63.88	7133.85 ± 36.60	7123.28 ± 56.67	7100.96 ± 29.53	7121.39 ± 37.71	7131.14 ± 44.32	7148.84 ± 51.13
CI	1.239 ± 0.119	1.228 ± 0.101	1.203 ± 0.108	1.193 ± 0.096	1.211 ± 0.079	1.197 ± 0.070	1.178 ± 0.075
HI	0.079 ± 0.012	0.069 ± 0.012	0.063 ± 0.013	0.060 ± 0.012	0.064 ± 0.015	0.067 ± 0.015	0.075 ± 0.014
GI	5.654 ± 0.660	5.799 ± 0.763	5.988 ± 0.811	6.002 ± 0.722	6.070 ± 0.771	6.173 ± 0.829	6.222 ± 0.862
NDC	0.973 ± 0.009	0.972 ± 0.011	0.968 ± 0.011	0.965 ± 0.011	0.969 ± 0.009	0.964 ± 0.011	0.966 ± 0.011
MUs	693.43 ± 103.08	664.12 ± 83.06	651.37 ± 75.67	654.69 ± 68.58	641.32 ± 47.86	645.67 ± 42.79	641.24 ± 48.84
MCSv	0.088 ± 0.012	0.127 ± 0.020	0.147 ± 0.019	0.151 ± 0.019	0.139 ± 0.016	0.120 ± 0.005	0.106 ± 0.008

Sd, standard deviation.

**Table 4:** The *P*‐value list of PTV dosimetric analysis (*n* = 10)

Collimator angles	*P*‐value												
	V100% (%)	V107% (cm^3^)	V95% (cm^3^)	V50% (cm^3^)	D2% (cGy)	D98% (cGy)	Dmean (cGy)	CI	HI	GI	NDC	MUs	MCSv
15°	0.508	0.123	0.241	0.285	0.139	0.285	0.139	0.575	0.085	0.169	0.677	0.037	0.005
30°	0.093	0.028	0.139	0.139	0.028	0.074	0.028	0.047	0.012	0.005	0.019	0.007	0.005
45°	0.114	0.028	0.139	0.114	0.022	0.037	0.007	0.037	0.005	0.013	0.005	0.047	0.005
60°	0.333	0.128	0.646	0.013	0.059	0.074	0.028	0.214	0.022	0.007	0.046	0.028	0.005
75°	0.169	0.310	0.878	0.013	0.386	0.114	0.114	0.203	0.053	0.005	0.007	0.093	0.005
90°	0.359	0.767	0.386	0.022	0.878	0.139	0.386	0.074	0.332	0.005	0.021	0.059	0.009

**Table 5:** The comparison of PTV dosimetric results (*n* = 10)

Collimator angles	*P*‐value												
	V100% (%)	V107% (cm^3^)	V95% (cm^3^)	V50% (cm^3^)	D2% (cGy)	D98% (cGy)	Dmean (cGy)	CI	HI	GI	NDC	MUs	MCSv
15°	–	–	–	–	–	–	–	–	–	–	–	L*	S**
30°	–	L*	–	–	L*	–	L*	S*	S*	H**	I*	L**	S**
45°	–	L*	–	–	L*	H*	L**	S*	S**	H*	I**	L*	S**
60°	–	–	–	H*	–	–	L*	–	S*	H**	I*	L*	S**
75°	–	–	–	H*	–	–	–	–	–	H**	I**	–	S**
90°	–	–	–	H*	–	–	–	–	–	H**	I*	–	S**

H, A higher value than a collimator angle of 0°; L, A lower value than a collimator angle of 0°; S, closer to optimal value than a collimator angle of 0°; I, more away from optimal value than a collimator angle of 0°.

*P≦ 0.05; **P < 0.01; −*P* > 0.05.

A collimator angle of 0° required the maximum number of MUs and a collimator angle of 90° required the minimum number of MUs. All other angles (15°, 30°, 45°, 60°, 75°, and 90°) required significantly fewer MUs than did the 0° angle. By definition, the MCS_V_ can have values in the range of 0–1. MCS_V_ = 1 indicates no modulation. When modulation increases, the MCSv decreases. The collimator angle of 0° exhibited the smallest value of MCS_V_, whereas all other angles had significantly higher values.

**Figure 3:**
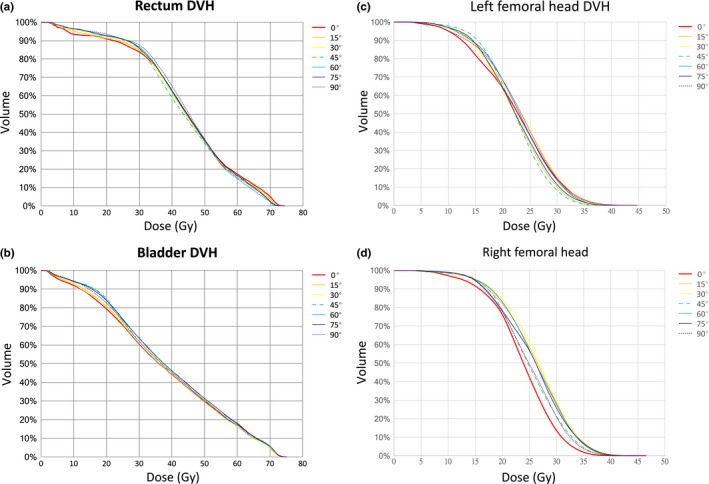
(a) Average dose–volume histogram of the rectum. (b) Average dose–volume histogram of the bladder. (c) Average dose–volume histogram of the left femoral head. (d) Average dose–volume histogram of the right femoral head.

The planning dose objectives of the rectum, bladder, and femoral heads were consistent with the constraints, and their dosimetric results are listed in Table 6. The *P* values and the comparison of the OARs dosimetric results are listed in Tables 7 and 8, respectively. Average DVH of the rectum and bladder are shown in Fig. 3. From the rectal dose observation, the highest values of maximum dose (D_max_) and V_60 Gy_ were obtained with a collimator angle of 0°. The lowest value of mean dose (D_mean_) was obtained with a collimator angle of 45°. From the bladder dose observation, the collimator angles of 75° and 90° had significantly higher values of D_mean_ than did the collimator angle of 0°. The values of D_max_ and V_60 Gy_ obtained at the other tested angles (15°, 30°, 45°, 60°, 75°, and 90°) were not statistically significantly different from those obtained at the collimator angle of 0°. For the femoral heads, none of the observed values (D_mean_, D_5%_, V_30 Gy_) were statistically significantly different from those obtained at the collimator angle of 0°.

**Table 6:** Dosimetric results of OARs (Mean ± SD, *n* = 10)

Parameters	Collimator angles						
	0°	15°	30°	45°	60°	75°	90°
Bladder							
Dmax (cGy)	7268.76 ± 187.64	7269.64 ± 140.77	7268.96 ± 175.86	7292.52 ± 190.84	7311.53 ± 171.80	7334.75 ± 132.18	7322.43 ± 132.95
Dmean (cGy)	3776.17 ± 1007.54	3786.40 ± 978.01	3826.00 ± 964.18	3885.19 ± 827.74	3887.81 ± 856.86	3923.18 ± 920.96	3915.33 ± 972.53
V_20 Gy_ (%)	79.37 ± 23.62	80.79 ± 23.11	81.09 ± 24.46	84.92 ± 14.66	84.23 ± 16.68	83.93 ± 18.89	82.58 ± 22.19
V_50 Gy_ (%)	29.44 ± 13.17	29.20 ± 13.22	30.20 ± 11.98	30.03 ± 12.81	30.31 ± 13.26	31.22 ± 13.79	31.07 ± 13.63
V_60 Gy_ (%)	17.01 ± 8.93	16.06 ± 8.74	16.96 ± 8.71	16.97 ± 8.65	16.77 ± 8.65	17.58 ± 9.26	18.04 ± 9.44
Rectum							
Dmax (cGy)	7280.30 ± 83.89	7219.72 ± 56.46	7199.70 ± 70.19	7140.57 ± 70.09	7167.78 ± 64.77	7189.62 ± 78.69	7189.89 ± 104.37
Dmean (cGy)	4393.03 ± 416.60	4386.66 ± 404.13	4384.76 ± 390.79	4362.80 ± 348.60	4410.35 ± 376.11	4437.61 ± 341.55	4460.03 ± 333.71
V_20 Gy_ (%)	90.99 ± 10.21	91.25 ± 9.72	91.75 ± 9.45	92.46 ± 9.71	92.38 ± 9.63	92.67 ± 9.83	93.60 ± 8.51
V_50 Gy_ (%)	35.25 ± 2.85	34.40 ± 3.87	34.14 ± 4.46	33.46 ± 3.86	34.46 ± 4.23	35.22 ± 4.31	35.72 ± 2.91
V_60 Gy_ (%)	16.97 ± 2.98	16.23 ± 2.75	14.85 ± 5.49	16.85 ± 4.05	15.23 ± 2.91	16.21 ± 2.80	14.40 ± 3.40
Right femur							
Dmean (cGy)	2671.6 ± 706.93	2748.94 ± 699.21	2790.44 ± 638.18	2646.51 ± 714.52	2831.69 ± 689.94	2840.94 ± 751.89	2621.10 ± 711.32
D_5%_ (cGy)	3333.84 ± 327.03	3376.52 ± 311.79	3489.07 ± 242.52	3255.79 ± 292.19	3382.65 ± 309.02	3422.57 ± 297.40	3253.34 ± 232.43
V_30 Gy_ (%)	21.37 ± 17.70	26.24 ± 19.54	28.30 ± 14.07	21.50 ± 18.24	29.91 ± 23.93	28.89 ± 21.89	13.85 ± 8.43
Left femur							
Dmean (cGy)	2317.26 ± 300.15	2266.52 ± 221.46	2270.98 ± 259.22	2252.73 ± 194.14	2357.24 ± 312.52	2212.56 ± 269.37	2243.07 ± 313.96
D_5%_ (cGy)	2870.92 ± 1066.31	3173.39 ± 270.81	3204.43 ± 245.71	3073.58 ± 212.93	3276.59 ± 285.59	3063.44 ± 335.32	3202.46 ± 389.94
V_30 Gy_ (%)	14.52 ± 12.26	11.02 ± 9.43	13.48 ± 12.30	7.91 ± 5.63	16.02 ± 12.75	9.92 ± 8.28	14.21 ± 12.21

OARs, organs at risk; sd, standard deviation.

**Table 7:** The *P*‐value list of OARs dosimetric analysis (*n* = 10)

Collimator angles	*P*‐value															
	Bladder					Rectum					Right femur			Left femur		
	D_max_ (cGy)	D_mean_ (cGy)	V_20 Gy_ (%)	V_50 Gy_ (%)	V_60 Gy_ (%)	D_max_ (cGy)	D_mean_ (cGy)	V_20 Gy_ (%)	V_50 Gy_ (%)	V_60 Gy_ (%)	D_mean_ (cGy)	D_5%_ (cGy)	V_30 Gy_ (%)	D_mean_ (cGy)	D_5%_ (cGy)	V_30 Gy_ (%)
15°	0.878	0.646	0.508	0.475	0.444	0.093	0.878	0.674	0.508	0.139	0.386	0.575	0.203	0.646	0.799	0.646
30°	0.878	0.575	0.799	0.799	0.959	0.017	0.959	0.260	0.214	0.093	0.203	0.169	0.241	0.386	0.508	0.721
45°	0.508	0.285	0.203	0.333	0.959	0.007	0.445	0.015	0.047	0.508	0.445	0.241	0.878	0.386	0.959	0.139
60°	0.203	0.074	0.203	0.114	0.575	0.007	0.445	0.011	0.445	0.013	0.114	0.575	0.203	0.507	0.386	0.508
75°	0.074	0.009	0.037	0.022	0.721	0.037	0.575	0.011	0.959	0.445	0.074	0.285	0.114	0.202	0.721	0.203
90°	0.241	0.028	0.110	0.059	0.285	0.047	0.169	0.008	0.646	0.013	0.575	0.386	0.203	0.646	0.767	0.959

OARs, organs at risk.

**Table 8:** The comparison of OARs dosimetric results (*n* = 10)

Collimator angles	*P*‐value															
	Bladder					Rectum					Right femur			Left femur		
	D_max_ (cGy)	D_mean_ (cGy)	V_20 Gy_ (%)	V_50 Gy_ (%)	V_60 Gy_ (%)	D_max_ (cGy)	D_mean_ (cGy)	V_20 Gy_ (%)	V_50 Gy_ (%)	V_60 Gy_ (%)	D_mean_ (cGy)	D_5%_ (cGy)	V_30 Gy_ (%)	D_mean_ (cGy)	D_5%_ (cGy)	V_30 Gy_ (%)
15°	–	–	–	–	–	–	–	–	–	–	–	–	–	–	–	–
30°	–	–	–	–	–	L*	–	–	–	–	–	–	–	–	–	–
45°	–	–	–	–	–	L**	–	H*	L*	–	–	–	–	–	–	–
60°	–	–	–	–	–	L**	–	H*	–	L*	–	–	–	–	–	–
75°	–	H**	H*	H*	–	L*	–	H*	–	–	–	–	–	–	–	–
90°	–	H*	–	–	–	L*	–	H**	–	L*	–	–	–	–	–	–

H, A higher value than a collimator angle of 0°; L, A lower value than a collimator angle of 0°; OARs, organs at risk.

*P ≦ 0.05; **P < 0.01; −*P* > 0.05.

## 4 DISCUSSION

All dual‐arc VMAT plans for prostate cancer with seven different collimator angles fulfilled the PTV dose requirements (V_100%_ ≥95%). In this study, the collimator angle of 0° had the highest value of V_107%_ (3.29 cm^3^), whereas the collimator angle of 45° had the lowest value (0.35 cm^3^). Additionally, the highest value of V_95%_ was observed with the collimator angle of 0° (231.55 cm^3^) and the lowest value of V_95%_ was observed with 45° (129.05 cm^3^). Regarding standardized metrics, the collimator angle of 0° exhibited inferior CI and HI values, whereas the 45° collimator angle exhibited relatively superior CI and HI values. Isa et al. also reported similar results that VMAT with a collimator angle of 45° provided superior PTV dose distribution, indicated by a high value of CI and low value of HI.^13^ Otto et al. indicated that IMRT delivery through MLC rotation improved dosimetric spatial resolution, thereby enabling superior target coverage.^12^ Bortfeld reported that VMAT with a collimator angle of 45° improved results, and this advantage was attributed to the fact that, with the 45° collimator angle, in parallel opposed beams the leaves of the MLC move in orthogonal directions.^16^ In addition, Otto explained that only a single leaf pair can be used to modulate intensity without MLC rotation, which yields inferior dose distributions.^14^

Although the PTV dose distribution obtained with a collimator angle of 0° was unsatisfactory, it had a lower GI value (5.65) than did all the other tested collimator angles. The highest GI value (6.22) was obtained with a collimator angle of 90°. The values of V_50%_ were significantly higher at collimator angles of 60°, 75°, and 90° than at a collimator angle of 0°. Because MLC rotation increases intermediate dose spillage, the NDC value obtained at a collimator angle of 0° was superior to those obtained at all other tested collimator angles. Badusha reported that the peripheral dose was significantly higher at a collimator angle of 70° than at 0°, and the difference in the dose was attributed to the increase in total area created by the jaws and higher spatial resolution in fluence map generation with a collimator angle of 70°.^22^

Generally, a high degree of complexity for IMRT is associated with parameters including large numbers of MUs, complex segment shapes, small segment apertures, and large numbers of segments. A complex plan requires a large number of MUs.^23^ In our investigation, the number of MUs required at a collimator angle of 0° was higher than that required with all other tested collimator angles. Additionally, the lowest value of MCS_V_ (0.088) was obtained without collimator rotation, which implies that the VMAT plan was highly complex. The numbers of required MUs are significantly lower at collimator angles of 15°, 30°, 45°, and 60° than those required at a collimator angle of 0°. At a collimator angle of 45°, the plan was less complex than that at 0° (MCS_V_ = 0.151). Radiotherapy plans, which are less complex, have higher probabilities of yielding accurate dosimetric results. Masi reported a significant positive correlation between the dose accuracy (gamma passing rates at 3%/3 and 2%/2 mm) of VMAT plans and the MCS_V_.^21^

In this investigation, the average mean dose to the rectum did not differ significantly among all the tested collimator angles. In addition, the values of D_max_ and V_60 Gy_ of the rectum were higher at a collimator angle of 0° than at all the other tested collimator angles. It was speculated that 0° collimator angle had inferior CI to result in more high‐dose area in rectum. Because of intermediate‐ and low‐dose spillage caused by MLC rotation, intermediate‐ and low‐dose areas were predominant in the rectum. According to parameters of the Lyman–Kutcher–Burman normal tissue complication probability model from four clinical series, Michaslski suggested that high doses are crucial for determining the risk of rectal toxicity.^24^ Thus, we can infer that MLC rotation does not increase the risk of late rectal toxicity. Because of the distensibility of the bladder, conducting robust dose–volume analyses were difficult. In this study, the mean bladder volume was 88.48 ± 55.37 cm^3^ (45.7–224.16 cm^3^). Some studies have reported that bladder “hotspots” are related to late bladder toxicity after external beam radiotherapy for prostate cancer.^25^, ^26^ In this study, because none of the tested collimator angles produced higher values of V_60 Gy_ for the bladder than did the 0° angle, MLC rotation was not considered to increase the risk of late bladder toxicity. For the left and right femoral heads, no statistically significant differences were observed for any of the tested angles.

## 5 CONCLUSION

Collimator angle selection could play a vital role in improving the plan quality of SIB‐VMAT for treating patients with prostate cancer. This study found that MLC rotation affects VMAT plan complexity and PTV dosimetric distribution. A collimator angle of 45° exhibited the optimal values of CI, HI, and MCSv among all the tested collimator angles. Some studies have reported that late side effects of the rectum and bladder are associated with high‐dose volumes. In this investigation, MLC rotation did not have statistically significantly higher values of V_60 Gy_ in the rectum and bladder than did the 0° angle. Based on the dose distribution of target and OARs, we thought a collimator angle of 45° was an optimal angle for the prostate VMAT treatment plan. The results of our study could serve as a guide for collimator angle selection with regard to PTV dosimetric distribution, plan complexity, and the sparing of OARs in prostate hypofractionated SIB‐VMAT planning.

## CONFLICT OF INTEREST

The authors have no conflicts of interest to disclose.
